# Ten-Year Single Institutional Analysis of Geographic and Demographic Characteristics of Patients Treated With Stereotactic Body Radiation Therapy for Localized Prostate Cancer

**DOI:** 10.3389/fonc.2020.616286

**Published:** 2021-02-25

**Authors:** Nima Aghdam, Michael Carrasquilla, Edina Wang, Abigail N. Pepin, Malika Danner, Marilyn Ayoob, Thomas Yung, Brian T. Collins, Deepak Kumar, Simeng Suy, Sean P. Collins, Jonathan W. Lischalk

**Affiliations:** ^1^ Department of Radiation Medicine, Beth Israel Deaconess Medical Center, Boston, MA, United States; ^2^ Department of Radiation Medicine, MedStar Georgetown University Hospital, Washington, DC, United States; ^3^ George Washington University School of Medicine, Washington, DC, United States; ^4^ The Julius L. Chambers Biomedical Biotechnology Research Institute, North Carolina Central University, Durham, NC, United States; ^5^ Perlmutter Cancer Center, Langone Medical Center, New York University, New York, NY, United States

**Keywords:** racial, disparities (health), machine learning, treatment burden, travel distance, prostate cancer, SBRT (stereotactic body radiation therapy)

## Abstract

**Objectives:**

Stereotactic Body Radiation Therapy (SBRT) offers definitive treatment for localized prostate cancer with comparable efficacy and toxicity to conventionally fractionated radiotherapy. Decreasing the number of treatment visits from over 40 to five may ease treatment burden and increase accessibility for logistically challenged patients. Travel distance is one factor that affects a patient’s access to treatment and is often related to geographic location and socioeconomic status. In this study, we review the demographic and geographic factors of patients treated with SBRT for prostate cancer for a single institution with over a decade of experience.

**Methods:**

Patient zip codes from one thousand and thirty-five patients were derived from a large, prospectively maintained quality of life database for patients treated for prostate cancer with SBRT from 2008 to 2017. The geospatial distance between the centroid of each zip code to our institution was calculated using the R package Geosphere. Characteristics for seven hundred and twenty-one patients were evaluated at the time of analysis including: race, age, and insurance status. To assess the geographic reach of our institution, we evaluated the demographic features of each zip code using US Census data. Statistical comparisons for these features and their relation to distance traveled for treatment was performed using the Mann-Whitney U test. Finally, an unsupervised learning algorithm was performed to identify distinct clusters of patients with respect to median income, racial makeup, educational level, and rural residency.

**Results:**

Patients traveled from 246 distinct zip codes at a median distance of 11.35 miles. Forty percent of patients were African American, 6.9% resided in a rural region, and 22% were over the age of 75. Using K-means cluster analysis, four distinct patient zip-code groups were identified based on the aforementioned demographic features: Suburban/high-income (45%), Urban (30%), Suburban/low-income (17%), and Rural (8%). For each of the clusters, the average travel distance for SBRT was significantly different at 11.17, 9.26, 11.75, and 40.2 miles, respectively (p-value: <0.001).

**Conclusions:**

Distinct demographic features are related to travel distance for prostate SBRT. In our large cohort, travel distance did not prevent uptake of prostate SBRT in African American, elderly or rural patient populations. Prostate SBRT offers a diverse population modern treatment for their localized prostate cancer and particularly for those who live significant distances from a treatment center.

## Introduction

Adoption of a new technology in cancer treatment is contingent upon efficacy, safety, and accessibility. The field of radiation oncology has historically been dominated by the concept of fractionation to optimize the therapeutic ratio. However, with the evolution of advanced imaging, precision radiotherapy delivery, and exquisite image guidance, our ability to reliably and precisely treat even moving targets has allowed for an unprecedented movement towards hypofractionation. While the oncologic efficacy and side effect profile of ultra-hypofractionated radiotherapy (UHF-RT) has been found to be comparable to other modes of radiation for localized prostate cancer, UHF- RT is currently offered to a minority of patients (1–3). Ultra-hypofractionated radiotherapy can be delivered in a five-fraction regimen, which has the potential to reduce treatment burden and cost, as well as improve accessibility to patients who may be burdened by fractionated radiation treatments delivered over nine weeks (4–6).

Health services utilization is partially determined by geographical disparity (7–9). Patients who live in areas with scarce healthcare options face greater barriers to accessing appropriate services and are required to travel long distances for cancer treatment (10). Specifically, patients from rural communities have diminished access to newer and novel treatments and practice changing clinical trials (11). Several studies have documented improved cancer outcomes for patients treated at centers with more specialized care (12–14). In general, travel distance to a major cancer center has been noted to contribute to slower adoption of new cancer treatment and poorer outcomes (12, 15). A recent study examining the National Cancer Database for prostate cancer revealed that travel distance may be a contributor to racial disparity for African Americans, Hispanics and other nonwhite races in the adoption of SBRT for treatment of localized prostate cancer (16). While the efficacy of SBRT continues to be evaluated in prospective clinical trials, the inequitable access to SBRT may prove detrimental. Given that nine weeks of daily conventionally fractionated radiation therapy may lead to greater financial toxicity for communities with lower income and decreased access due to geographic disparity, a hypofractionated regimen offers an excellent treatment option to patients with limited access without overwhelming logistical challenges.

To this end, we sought to review a decade of experience at a comprehensive cancer center which was an early adopter of SBRT for localized prostate cancer. Using a large institutional database, we analyzed the geographic and demographic features of our patient population, the utilization of prostate SBRT defined by geodemographic clusters based on zip code, and associated census data points using a machine learning algorithm.

## Methods

**Figure 1:**
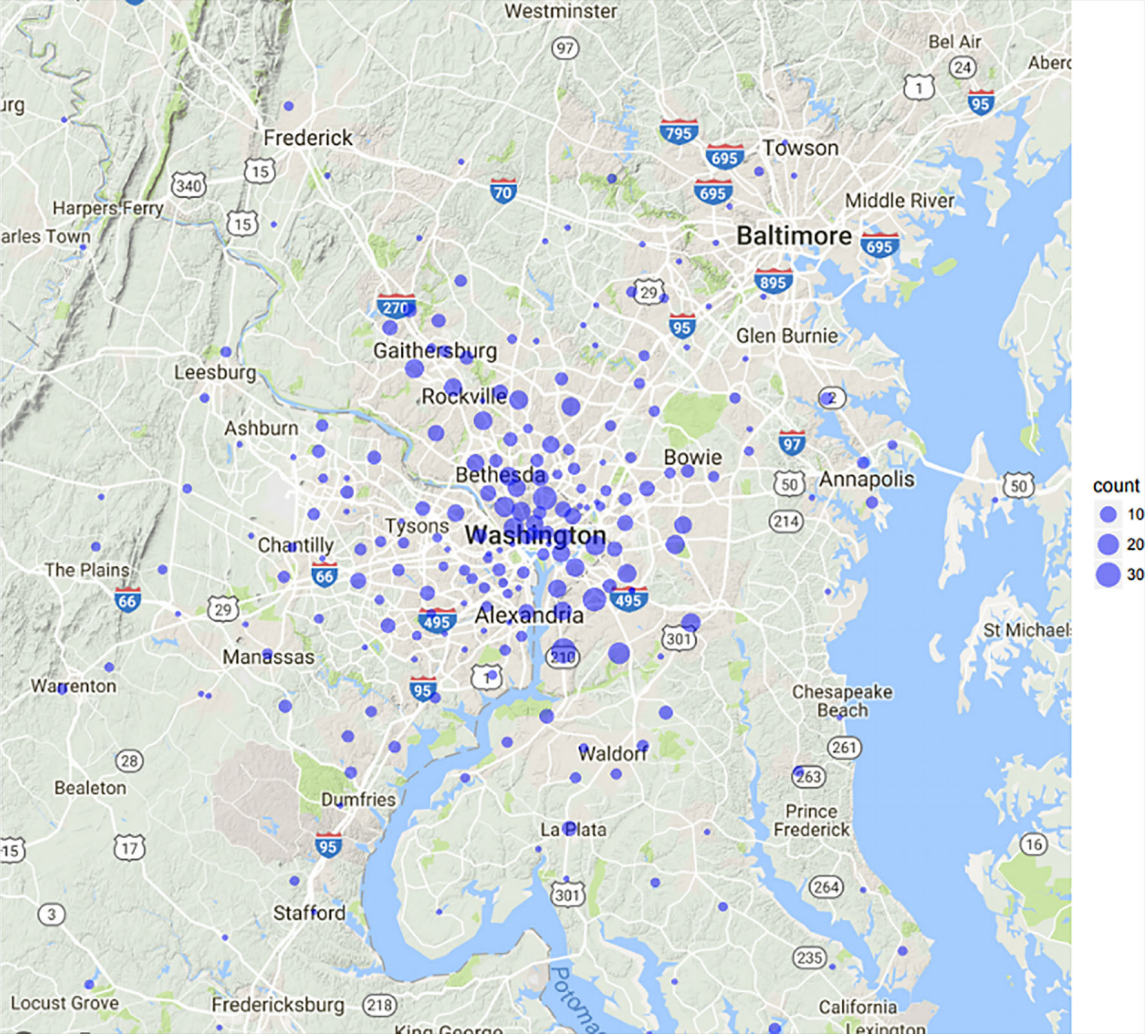
Map patient zip codes.

From January of 2008 to December of 2017, 1,035 patients with localized prostate cancer were treated at Medstar Georgetown University Hospital with five fraction SBRT or an SBRT boost and supplemental pelvic IMRT. Given that a portion of patients traveled long distances across the United States a threshold for outliers was developed using the Tukey method (**Figure 1**). Subsequently, full records for 923 patients with localized prostate cancer at Georgetown University Hospital were analyzed. Of these, 725 patients were treated with SBRT monotherapy and 198 with an SBRT boost in addition to conventionally fractionated pelvic radiotherapy. Treatment methods have been described elsewhere (17) but briefly; one week after placement of 4 to 6 gold fiducial markers in the prostate, patients underwent a CT simulation of the pelvis. The bladder, prostatic urethra, membranous urethra and rectum were contoured by a single treating radiation oncologist (SPC). Inverse planning was generated with a prescription dose of 35 to 37.25 Gy in five fractions using 6-MV photons calculated on MultiPlan software (Accuray Inc., Sunnyvale, USA). Patients who received supplemental IMRT were treated with robotic SBRT (19.5 Gy in three fractions to the prostate) followed by fiducial-guided IMRT. Patients were initiated on IMRT treatment the week following SBRT. Daily doses of 1.8 Gy were delivered 5 days a week to a total dose of 45–50.4 Gy in 25–28 fractions. Dose volume histograms were constructed to meet clinically established dose objectives and constraints for OARs. Treatment was delivered using the CyberKnife robotic radiosurgical system (Accuray Inc., Sunnyvale, CA, USA). Fiducial tracking using continuous orthogonal x-rays was employed to account for intrafractional target motion.

The patient characteristics were derived from a prospectively maintained quality of life IRB (IRB#: 2009-510) approved institutional trial. Patient zip codes were extracted from the hospital billing database and the US Census database was accessed and linked with patient zip codes. The zip code data points included: median income, proportion of African American and rural residents, education level and proportion of un-insured patients. The geospatial distance between the centroid of each zip code to our cancer center was calculated using the R package Geosphere (CRAN, Vienna, Austria). These distances did not necessarily represent driving distance but rather as the crow flies. Statistical comparisons for these features and their relation to distance traveled for treatment was performed using the Mann-Whitney U test. After standardizing data, an unsupervised learning algorithm called K means clustering using SPSS version 23 (IBM Corp., Armonk, NY) was performed to identify distinct clusters of patients with respect to median income, racial makeup, educational level and rural residency (18). Distance traveled from each cluster was reported in miles. Differences in demographics for each zip code were interrogated to identify the chief discriminant of clusters. Google maps data was used to generate images and zoom levels meant to capture the Maryland and Virginia area from which our cohort lives.

## Results

In our cohort, the median age was 69 (range 48–92). Self-reported race was 46% Caucasian, 48% African American, and 6% Other. The majority of patients presented with intermediate risk prostate cancer (55%) per D’Amico criteria, followed by high risk (25%) and low risk (20%) disease. Mean and median travel distance to Georgetown University Hospital was 16.8 and 11.4 miles (0.44–222.2). Additional patient characteristics are presented in **Table 1**.

**Table 1:** Selected patient characteristics.

Median Age (range)	Number (%)
69 (48-92)	
**Race**	
White	**423** (46%)
Black	**442** (48%)
Other	**58** (6%)
**Risk group (D’Amico)**	
Low	**185** (11%)
Intermediate	**511** (63%)
High	**228** (26%)
**Treatment Modality**	
SBRT monotherapy	**725** (79%)
SBRT boost	**198** (21%)
**Travel Distance (miles)**	
11.8 (0.44-222.2)	
Total Patients: 923	

Bold is number of patients.

**Figure 2:**
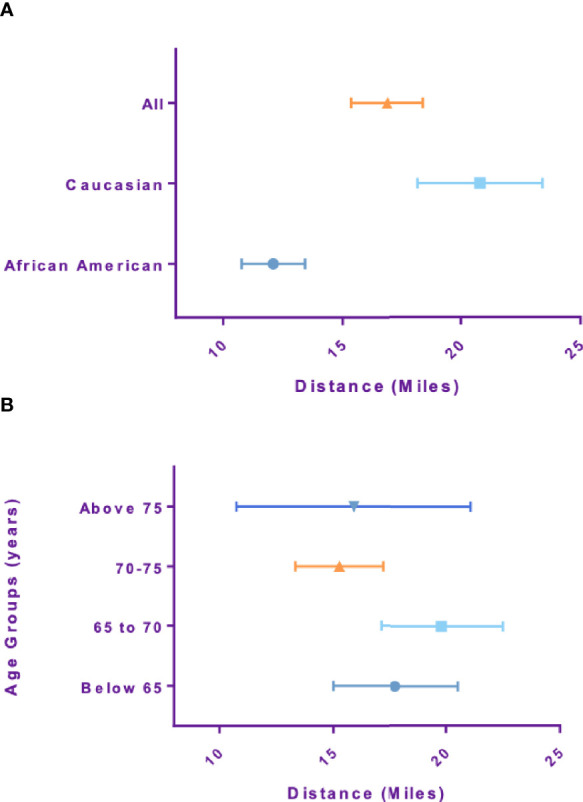
Travel distance stratified by **(A)** race and **(B)** Age.

Mean Travel distance for African American patients was 12.5 miles, which was significantly lower than Caucasian patients, 20.6 miles (p-value<0.001). Travel distance for patients older than 75 was 15.6 miles and not significantly different compared to patients younger than 65 which was 17.8 miles (p-value=0.19) (**Figures 2A, B**). Travel distance for patients with high risk disease was 13.9 miles, significantly lower than those with intermediate and low risk disease at 17.9 miles (p-value=0.014). Patients treated with supplemental IMRT traveled a shorter distance of 13.7 miles compared to monotherapy patients at 17.7 miles (p-value=0.017).

**Figure 3:**
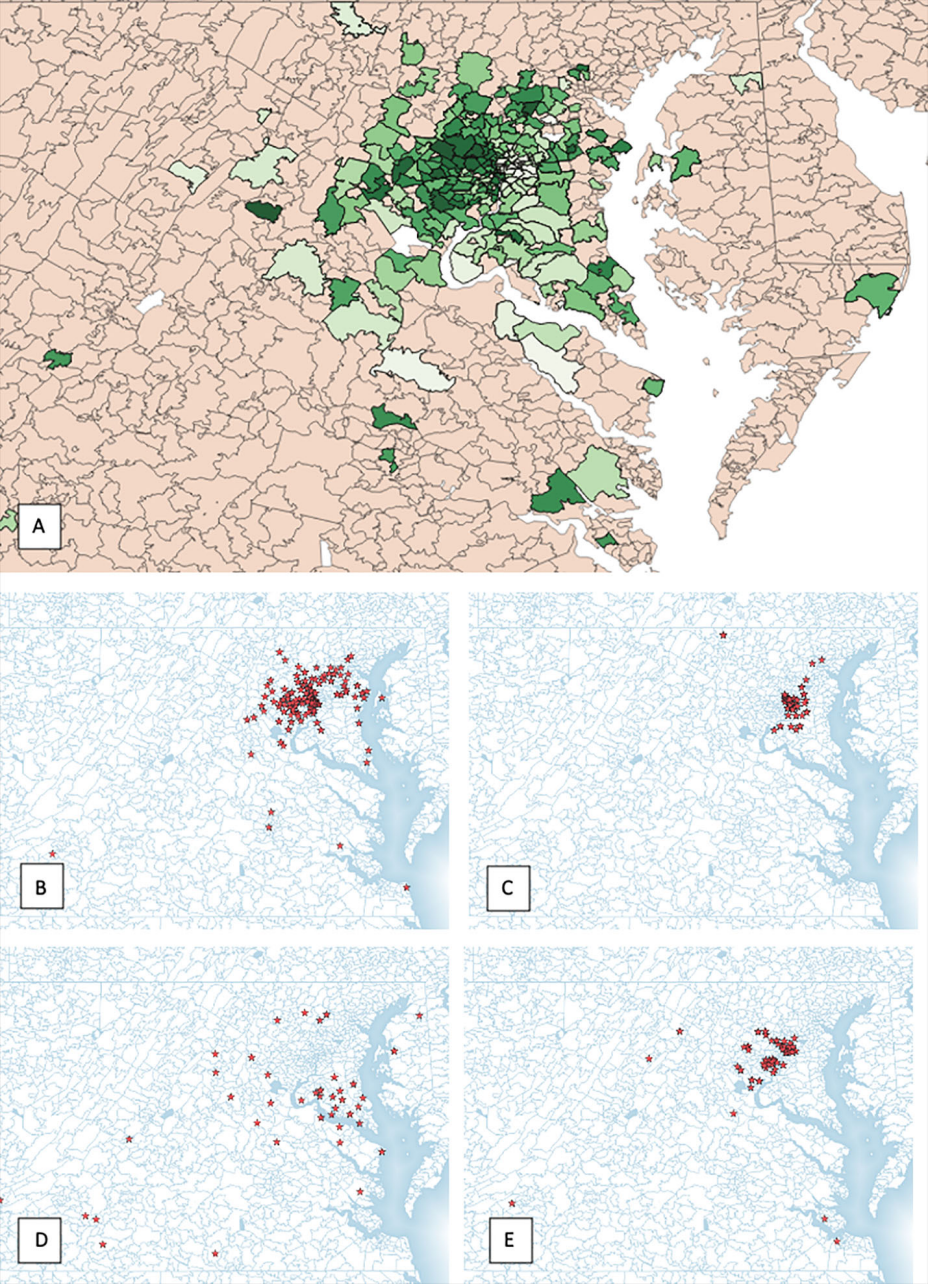
**(A)** All patient zip codes mapped, **(B)** urban cluster, **(C)** suburban/high income cluster, **(D)** suburban/low income cluster and **(E)** rural cluster.

**Figure 4:**
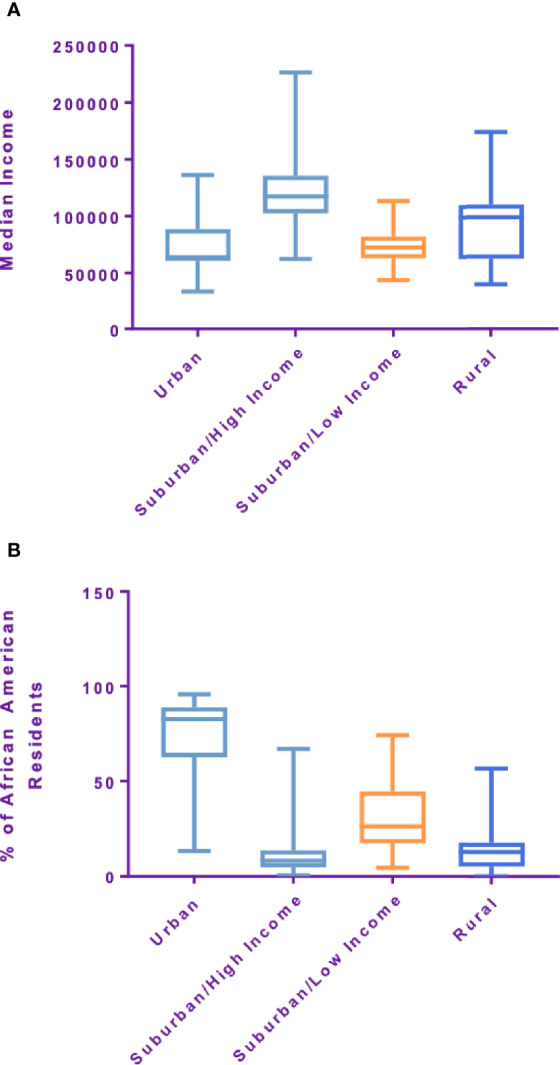
**(A)** median income by cluster and **(B)** racial makeup by cluster.

Within a 222 mile radius of the hospital 246 distinct zip codes were identified. The median income of identified zip codes was $107,170 ($34,739–226,386). The median percentage of African American residents in the zip codes analyzed was 21.39% (0%–100%). Approximately 6.9% of zip codes analyzed were considered rural based on rural residency (**Table 2**). Using an unsupervised K-means clustering algorithm which included the above characteristics as well as percentage uninsured and percentage high school graduates within each zip code, four distinct clusters with similar demographic features were identified (**Figure 3**). The clusters were characterized based on rural residency, African American population, and income, and are referred to as: Urban, Suburban/high income, Suburban/low income and Rural clusters. Mean travel distance for the urban cluster was 9.26 miles, compared to 11.17 miles for Suburban/high income, 11.75 miles for Suburban/low income and 40.2 miles for the rural cluster. For each of the clusters, the average travel distance for SBRT was significantly different (p-value: <0.001). Incomes differed significantly between clusters with the urban clusters having the lowest income with a median income of $63,000 per household and the suburban/high income cluster having highest median income at $125,000 dollars per household. Racial make-up of each cluster differed significantly, with the urban cluster having an 82% African American population compared to suburban/high income at 9% (**Figure 4**).

**Table 2:** Selected patient demographics.

Demographic Feature Median (Range)	
**Income** (USD)	
	**107,170** (34,739-226,386)
**% African American Residents**	
	**21.39%** (0%-100%)
**% Rural Residents**	
	**6.9%** (0%-100%)
**% Un-Insured residents**	
	**6.5%** (0-30.8%)

## Discussion

SBRT for localized prostate cancer is increasingly offered as a treatment option that may reduce treatment related burden compared to conventionally fractioned EBRT. This treatment regimen has the potential advantage of being more accessible to patients than conventionally fractionated EBRT. We reviewed the demographic and geographic factors of patients treated with SBRT for localized prostate cancer at a single institution with over a decade of prostate SBRT experience. To our knowledge, this is the first study that examines patient utilization of prostate SBRT based on sociodemographic clusters derived from a machine learning algorithm. In this study, patient age, zip code, and race as well as US census data tied to patient zip codes were entered into an unsupervised K-means clustering algorithm to categorize geographical and demographical clusters.

Studies in the past have demonstrated travel distance as one of the factors that affects a patient’s access to treatment and is often a consequence of other sociodemographic factors (15, 16). Mahal et al. identified distinct demographic features that correlate with travel distance specifically for prostate SBRT such as race, income and rural residency (16). In this study, travel distance was found to be a function of race, income and rural residency, consistent with findings from Mahal and colleagues (16). In our study, Caucasian patients traveled significantly further for their treatment than African American patients. However, it is noteworthy that the racial make-up of our study population for the most part, mirrored that of the general population of the community surrounding our institution, the District of Columbia (43% AA vs 46% AA, 53% Caucasian vs 46% Caucasian, respectively) (19). These findings imply that travel distance may be an incomplete proxy for access to care. Further, we found that our patients came from a relatively balanced mix of socio-demographic backgrounds with the majority coming from Urban, Suburban/low income and Rural clusters but not Suburban/high income. This finding is interesting as the clusters had significantly different median travel distances. Importantly, despite an almost 4-fold greater travel distance compared to their Urban and Suburban counterparts (40.2 vs 10.72 miles), patients from the rural cohort were able to access this treatment regimen. We suspect that this greater geographic accessibility is due to the reduced number of treatment sessions associated with SBRT as compared to conventionally fractionated EBRT. Interestingly, we found that the distance traveled for patients receiving SBRT boost and supplemental IMRT was significantly shorter than patients who were treated with SBRT alone. This is likely a result of the significantly higher number of treatments needed for the SBRT/IMRT combination, resulting in a greater treatment burden and lower geographic accessibility.

Travel distance stratified by risk group demonstrates a significantly lower distance traveled for individuals with high risk disease than with intermediate or low risk groups. Despite this, there was no significant difference in travel distance for individuals requiring a boost. This is likely because boost protocols include high risk and intermediate risk patients.

In prior studies, patients who are African American, under the age of 65, those with low income and/or with a low education level were identified as more likely to experience reduced access to cancer treatment (20, 21). In this study, we found significant utilization of prostate SBRT from African American, elderly, low income and rural communities. This suggests that utilization of SBRT for prostate cancer may improve access to patient populations that have historically faced a disproportionate barrier in treatment of their cancer. Decreasing the number of treatments to five may ease treatment burden and increase accessibility for logistically challenged and socioeconomically disenfranchised patients. Furthermore, SBRT may be an option for some patients to reduce financial toxicity related to their cancer care while achieving excellent disease specific outcomes (1).

Potential limitations of our study include the use of indirect patient characteristics based on US Census data, the exclusion of patients who traveled very long distances to receive treatment, the possibility of inaccuracies in the US Census data used and changes in the demographics of patients who utilized SBRT over the study period. Future directions include using artificial intelligence derived clusters to study disease specific end-points, patient reported outcomes and possibly to identify disparities that were missed when only considering single variables.

## Conclusion

In this study, we examined distinct demographic features and their relationship with travel distance for prostate SBRT. Notably, travel distance did not prevent the uptake of this new technology for our African American, elderly or rural patients. Hence, prostate SBRT is a modern treatment modality that a diverse population can access, particularly for those who live significant distances from a treatment center. This is likely secondary to shortened treatment time offered by this technology compared to conventionally fractionated radiotherapy.

## Data Availability Statement

The raw data supporting the conclusions of this article will be made available by the authors, without undue reservation.

## Ethics Statement

The studies involving human participants were reviewed and approved by Georgetown University Hospital IRB#: 2009-510. The patients/participants provided their written informed consent to participate in this study.

## Author Contributions

NA is lead author who participated in manuscript drafting, table/figure creation, and manuscript revision. MC, EW, and AP aided in table/figure creation and manuscript drafting and revisions. MA, TY, and MD assisted in data collection, organization, and manuscript revisions. SS, SC, DK, BC, and JL are senior authors who aided in drafting the manuscript and manuscript revision. JL is the corresponding author who initially developed the concept, drafted, and revised the manuscript. All authors contributed to the article and approved the submitted version.

## Funding

This work was supported by NIH Grant P30CA051008 and grant R01MD012767 from the National Institute on Minority Health and Health Disparities (NIMHD), NIH to DK and SC.

## Conflict of Interest

SC, BC, and JL serve as clinical consultants to Accuray Inc.

The remaining authors declare that the research was conducted in the absence of any commercial or financial relationships that could be construed as a potential conflict of interest.
